# Hematopoietic Cancer Cell Lines Can Support Replication of Sabin Poliovirus Type 1

**DOI:** 10.1155/2015/358462

**Published:** 2015-02-28

**Authors:** Dinja Oosterhoff, Gerard van de Weerd, Gerco van Eikenhorst, Tanja D. de Gruijl, Leo A. van der Pol, Wilfried A. M. Bakker

**Affiliations:** ^1^Institute for Translational Vaccinology, P.O. Box 450, 3720 AL Bilthoven, Netherlands; ^2^Department of Medical Oncology, VU University Medical Center, P.O. Box 7057, 1007 MB Amsterdam, Netherlands

## Abstract

Viral vaccines can be produced in adherent or in suspension cells. The objective of this work was to screen human suspension cell lines for the capacity to support viral replication. As the first step, it was investigated whether poliovirus can replicate in such cell lines. Sabin poliovirus type 1 was serially passaged on five human cell lines, HL60, K562, KG1, THP-1, and U937. Sabin type 1 was capable of efficiently replicating in three cell lines (K562, KG1, and U937), yielding high viral titers after replication. Expression of CD155, the poliovirus receptor, did not explain susceptibility to replication, since all cell lines expressed CD155. Furthermore, we showed that passaged virus replicated more efficiently than parental virus in KG1 cells, yielding higher virus titers in the supernatant early after infection. Infection of cell lines at an MOI of 0.01 resulted in high viral titers in the supernatant at day 4. Infection of K562 with passaged Sabin type 1 in a bioreactor system yielded high viral titers in the supernatant. Altogether, these data suggest that K562, KG1, and U937 cell lines are useful for propagation of poliovirus.

## 1. Introduction

Vaccines are pharmacological formulations that incorporate the disease-causing agent or an antigen derived from this agent, which are capable of inducing an immune response once administered to a healthy individual, without causing the disease itself. Licensed vaccines can be divided into viral and bacterial vaccines, and viral vaccines can be further classified into four categories: live attenuated viruses, inactivated viruses, subunit vaccines, and virus-like particles. For the production of the first two categories, large amounts of viral particles are needed, and most of these viral vaccines are produced by infecting susceptible cell lines. Since there is no standard cell line that can be used for the replication of every virus, a whole panel of different cell lines has been used for vaccine production processes throughout the years. Cell lines that have historically often been used for the production of viral vaccines are MRC-5 and WI-38 [1, 2]. These two cell lines are human diploid cell lines derived from fetuses, and these cells were used for the manufacture of a number of vaccines, for example, hepatitis A, polio, and rubella [3–5]. Diploid cell lines have a finite lifespan and in these cell lines the chromosomes are paired. Often these cells retain many characteristics of the cell types from which they originate. The disadvantage of diploid cell lines lies in the fact that the cells can only be cultured for a limited number of passages before the cells die of senescence. In general, diploid cells grow as adherent cells and require serum-containing growth media to grow efficiently. The major benefit of diploid cells is the fact that the cells are nontumorigenic and therefore are considered safe to use for the production of vaccines (reviewed by Hayflick et al. [6]). Given the high demand of vaccines and the restrictions associated with the use of diploid cell lines, in the last decades, continuous cell lines were introduced in vaccine production processes. From a vaccine production point of view, the characteristic of continuous growth is beneficial, since such cells have the potential for an infinite lifespan, and characterized and approved master and working cell banks can be established. A thorough understanding of the cell substrates with respect to identity, stability, purity, tumorigenicity, and the presence of adventitious and endogenous agents is, however, essential for the production of quality assured vaccines [7]. The first continuous cell line approved for the production of vaccines was the Vero cell line, originating from African green monkeys and developed at the Chiba University in Japan. The mechanism of immortalization of Vero cells is unknown. It has been described that Vero cells at passages 140 to 165 are not tumorigenic in immunocompromised mice [8–10] and at those passages Vero cells are currently used for the manufacturing of viral vaccines. A recent paper, however, concluded that the transition from nontumorigenic to a tumorigenic phenotype of Vero cells did not occur until passage 185 [11]. Vero cells have, over the years, proven to be safe, since millions of vaccine doses produced on Vero cells have been given to healthy individuals. A major advantage of Vero cells is that the cells are sensitive to infection with many different viruses [12], meaning that Vero cells can be used for the production of a number of different vaccines [13–16]. This wide infectivity may be the result of a defective antiviral interferon response of susceptible cells, which was demonstrated in general for cells that are permissive for poliovirus replication [17]. However, not all viruses are capable of replicating on Vero cells and the consensus is that the current repertoire of cell substrates is inadequate for the manufacture of certain types of (new) vaccines. To address this limitation, the Vaccines and Related Biological Products Advisory Committee Meeting (VRBPAC) recognized in 2012 that (human) tumor-derived cell lines could be an important addition to the repertoire of cell substrates for the production of viral vaccines (http://www.fda.gov/downloads/AdvisoryCommittees/CommitteesMeetingMaterials/BloodVaccinesandOtherBiologics/VaccinesandRelatedBiologicalProductsAdvisoryCommittee/UCM319573.pdf). In some cases, even the only susceptible cell available to propagate specific viruses for which vaccines are needed could be tumor cells. Therefore, currently several tumor cells lines are being explored for their capacity to propagate viral vectors, like the Madin-Darby canine kidney (MDCK) cell line [18], HeLa cell line [19, 20], and the PER.C6 cell line of which the latter was immortalized by transfection with adenoviral E1 proteins [21]. At the present time though only a limited number of vaccines that were produced in tumorigenic cell lines have entered clinical trials or were registered [22–26].

Overall, it can thus be stated that the regulatory opinion on cell lines that are used for the production of viral vaccines has changed radically at the last 30 years with respect to the risks and benefits of immortalized tumorigenic cell lines [7, 26]. This is most likely also due to the development of techniques that can detect adventitious viruses or host cell DNA in vaccines with a very high sensitivity [27]. In the future, a potential use of human tumor cell lines for the production of viral vaccines can be foreseen.

A characteristic of viruses is that viruses evolve, and due to mutations in their genetic material or recombination with other viruses, outbreaks of dangerous and potential lethal new viruses can occur. In these cases, fast development of vaccines is essential for global health. It would be of great importance if cell lines that are needed for the production of such vaccines could be selected upfront, at the start of a viral outbreak, based on scientific understanding instead of trial and error. In this study, we have made a start with the characterization of human tumor cell lines and their capacity to support viral replication. Five human cell lines, capable of growing in suspension and often used in research, but not currently qualified for vaccine production purposes, were selected, HL60, K562, KG1, THP-1, and U937 [28–32]. These cell lines are all cancer cell lines and originally derive from patients with leukemia or lymphoma. All different cell lines originate from blood cells that were abrogated in their development at different promonocytic stages. Depending on the mix of cytokines and/or growth factors added to the cells, the cells can differentiate towards several end-stages. Since the stage of differentiation of a cell can have an effect on susceptibility of the cell for replication of specific viruses, this could be an interesting feature of the selected cell lines [33–35]. Possibly, the cells can become infected as progenitor cells, whereas infection is not possible when the cells are differentiated or vice versa. Interestingly, K562 cells are currently used as a vaccine for patients with lung cancer. Irradiated K562 cells, transfected with the gene encoding GM-CSF and CD40 ligand, were mixed with allogeneic tumor cells and this vaccine was tested in a phase II trial in lung cancer patients [36].

The overall aim of this study is to generate data that will facilitate decision making on which substrate may be the best for the production of novel vaccine strains. As a first step for this, we investigated whether poliovirus, as a representative of the Picornaviridae family of viruses, can be propagated in the human suspension cell lines.

## 2. Materials and Methods

### 2.1. Cells and Culture Conditions

The human hematopoietic progenitor tumor cell lines HL60, U937, K562, KG1, and THP-1 were cultured in IMDM (HL60, U937, K562, and KG1) (Invitrogen) or RPMI (THP-1) supplemented with 10% fetal bovine serum (FBS, PAA) and 10 U/mL penicillin-streptomycin (Gibco) at 37°C in a 5% CO_2_ humidified atmosphere. The Vero cell line was taken along as a positive control cell line and these cells were cultured in Virus Production Serum-Free Medium (VP-SFM, Invitrogen) supplemented with 2 mM glutamine (Life Technologies) at 37°C in a 5% CO_2_ humidified atmosphere. CHO suspension cells, which served as a negative control, were cultured in Ex-cell 302 medium (Sigma) in shaker flasks (Corning) gently rotating at 50–100 rpm at 37°C in a 5% CO_2_ humidified atmosphere.

To determine whether K562 cells were capable of growing in bioreactors, Cultibags (Sartorius Stedim) were inoculated with cells at a concentration of 0.3 × 10^6^ cells/mL in 1 L of culture medium. Cultibag cultures were performed at 37°C, dissolved oxygen concentration of 50%, pH 7.2 at a constant rocking speed of 10 rpm, and an angle of 7°.

### 2.2. Adaptation of Sabin Poliovirus Type 1 to the Hematopoietic Tumor Cell Lines

To serially passage Sabin poliovirus type 1 on the hematopoietic cell lines, 1 × 10^6^ cells were infected in T25 flasks with an MOI of 1. Cells and supernatants were harvested if full cytopathic effect (CPE) was observed or after 1 week of culture. After freeze-thawing three times to lyse the cells, samples were centrifuged at 3000 rpm to remove cellular debris and half of the supernatant was used to reinfect new hematopoietic cells. This was repeated for 4 times to allow the virus to adapt to the new cell lines in 5 passages. In the last infection round, 1 × 10^7^ cells in a T175 flask were infected and samples were harvested at day 3 (VERO and U937) or day 6 to obtain small viral stocks that were used for subsequent experiments.

### 2.3. Virus Kinetics

To determine replication kinetics, the susceptible tumor cell lines were infected with Sabin poliovirus type 1 from the parental virus or virus that was passaged for 5 times on the hematopoietic cell lines at MOI 1 or MOI 0.01, and samples of the supernatant and cellular lysates were harvested at different time points. After harvesting, the supernatant was centrifuged at 1000 rpm for 4 minutes to remove cells floating in the medium that did not release their progeny virus yet, and this fraction was added to the cellular fraction. Cells still attached to the flask were harvested by scraping the cells from the flask with a cell scraper (Becton Dickinson) in PBS. After centrifugation at 1000 rpm, PBS was removed and the cell pellet was resuspended in 1 mL of PBS. The cellular samples were freeze-thawed three times to release virus from the cells, and after centrifugation at 3000 rpm supernatant was transferred to a new tube. The virus titer in all the samples was determined by end-point titration on Vero cells.

K562 cells grown in Cultibags were infected at an MOI of 0.01 with passaged Sabin poliovirus type 1 when cells reached a concentration of 1.2–1.5 ×10^6^ cells/mL, and samples of the culture medium were taken at days 3, 4, 5, and 6 after infection. Samples were filtered (0.22 *μ*M filter) to remove viable cells and the virus titer and D-antigen levels in these samples were determined.

### 2.4. Virus Titration Assay

To determine the virus titer of Sabin poliovirus type 1 that replicated in the different cell lines, the virus titer was determined by end-point titration. In short, adherent Vero cells were seeded at a concentration of 1 × 10^4^ cells/100 *μ*L in 96-well flat bottom plates in M199 medium containing 10% serum. Serial 10-fold dilutions were prepared from cellular lysates or supernatant from hematopoietic cells infected with Sabin poliovirus type 1 in serum-containing M199 medium, and 50 *μ*L of these dilutions was added to 6 separate wells. After 7 days, all the wells were scored for the presence or absence of cytopathic effects (cpe), and the virus titer was determined using the Reed and Muench method, thereby calculating the 50% cell culture infective dose (CCID50)/mL. To calculate the plaque forming unit titer (pfu) from the CCDI50 value, the CCID50 was multiplied with 0.69 to generate the pfu titer/mL. To be able to compare the number of infectious Sabin type 1 polioviruses in the supernatant samples with the cellular lysate samples, the total amount of pfu in the samples was determined by multiplying the pfu/mL titer with the total amount of supernatant or cellular lysate that was harvested.

### 2.5. D-Antigen ELISA

To determine the presence of D-antigen in the virus samples, a sandwich ELISA was performed as described previously [37]. Briefly, plates were coated with an anti-Sabin type 1 caprylated bovine antiserum diluted 1 : 1600 in PBS (Gibco) overnight at 4°C. After washing, samples were added for 30 minutes at 37°C. After washing, a mixture of a Sabin type 1 specific mouse monoclonal antibody and an HRP-labeled goat anti-mouse antibody were added for 30 minutes at 37°C. After four washing steps, the signal reagent HighLite was added and the emitted light was detected with a luminometer.

### 2.6. Antibodies and Flow Cytometry

To determine the expression of viral receptors on the different cell lines, FACS analyses were performed. PE-labeled antibodies directed against human CD155 (eBioScience), CD54 (BD Pharmingen), and CAR (Millipore) and FITC-labeled anti-human CD81 (BD Pharmingen) were used for flow cytometric analyses, with FITC- or PE-labeled IgG1 antibodies (BD Pharmingen) as controls. Antibody staining was performed in PBS supplemented with 0.1% BSA and 0.02% sodium-azide for 30 min at 4°C. After washing of the cells, the stained cells were analyzed on a Guava FACS (Merck Millipore) using Cell FlowJo software.

## 3. Results

### 3.1. Efficient Replication of Sabin Poliovirus Type 1 in U937, K562, and KG1 Cells

**Figure 1:**
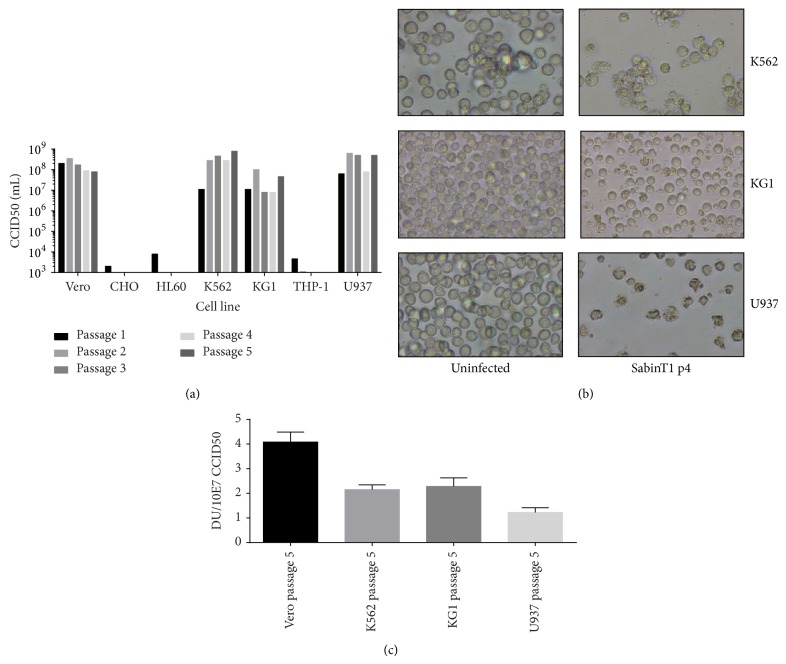
Viral titer in CCID50 value (a) or D-antigen level (c) of Sabin poliovirus type 1 per mL during serial passaging of Sabin poliovirus type 1 in hematopoietic tumor cell lines. Vero cells were used as a positive control cell line, whereas CHO cells served as a negative control. For the first infection, cells were infected with an MOI of 1, and cells together with supernatant were harvested after 3–6 days. After freeze-thawing of the material, half of it was used to reinfect fresh cells. This was repeated for 4 times. The virus titer in the 5 viral passages was determined by titration assay and the results are shown in (a). In (b) light microscopic images of uninfected or cells infected with Sabin poliovirus type 1 from passage 4 at day 6 (KG1 and K562) or day 3 (U937) are shown, whereas in (c) the D-antigen levels/10^7^ CCID50 for the fifth viral passage is shown.

To determine whether hematopoietic cell lines can support replication of Sabin poliovirus type 1, cells were infected with an MOI of 1 and cells together with supernatant were harvested at day 3 (for all virus passages in Vero cells and for passages 3–5 on U937 cells) or day 6 after infection. After lysis and centrifugation of the cells, half of the supernatant was used for the reinfection of new cells. This procedure was repeated for 4 more times, and virus derived from passage 5 was used for additional experiments. In the samples derived from all 5 passages, the virus titer (CCID50/mL) was determined by a virus titration assay. As shown in Figure 1(a), replication was efficient in the control cell line Vero, grown in serum-free medium, yielding high titers of more than 1 × 10^7^ CCID50/mL in all 5 passages. In CHO cells, Sabin poliovirus type 1 could only be detected in the first passage, and this is most likely due to the fact that cells were not washed after primary infection, suggesting that the virus titer is the result of virus that remained present in the culture medium, which was unable to infect the cells. In samples from passages 2–5 of Sabin poliovirus type 1 added to CHO cells, as expected no virus could be detected. A comparable pattern was observed after infection of THP-1 and HL60 cells with Sabin poliovirus type 1, suggesting that these two cell lines were refractory for the virus. The K562 and U937 cell lines were highly permissive for Sabin poliovirus type 1, resulting in high virus titers in all 5 viral passages. KG1 cells were also fully permissive for Sabin poliovirus type 1, but the amount of virus obtained after infection of KG1 cells was slightly lower compared to Sabin poliovirus type 1 that replicated in the other permissive cell lines. Microscopically it was observed that U937 cells infected with virus from passages 0–2 did not show clear cytopathic effects (cpe) after 6 days of culture, whereas cells infected with virus from passages 3, 4, and 5 were harvested after 3 days because full cpe was observed. A photographic overview of cells infected with virus from passage 4 is shown in Figure 1(b). Also, in K562 cells, clear cpe was visible at day 6 after infection. The amount of virus produced after replication of Sabin poliovirus type 1 in K562 cells did seem to increase after the first passage, suggesting that replication of the passaged virus is more efficient than replication of the parental virus.

To be able to compare the D-antigen level per virus, the specific D-antigen level per infectious virus particle was calculated for the fifth viral passage in the permissive cell lines and is shown in Figure 1(c). For Vero cell derived Sabin poliovirus type 1, the D-antigen per infectious virus particle was 4 DU/10^7^ CCID50. In general, it was observed that passaged virus had a D-antigen level per virus that was slightly lower compared to virus replicated in Vero cells, varying between 1.2 and 2.3 DU/10^7^ CCID50 depending on the cell line used.

### 3.2. All Hematopoietic Tumor Cell Lines Express CD155, the Poliovirus Receptor

**Figure 2:**
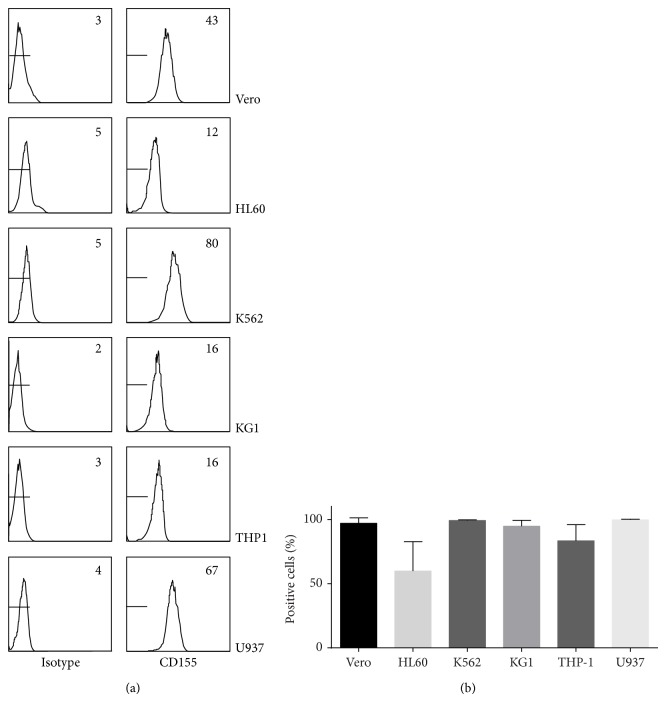
Surface expression of CD155, the receptor for poliovirus, on Vero, CHO, and hematopoietic tumor cell lines using FACS analysis. In (a) CD155 expression from a representative experiment is shown. In the right upper corner the mean fluorescence intensity levels are shown. In (b) the mean percentage of CD155 expressing cells ± SD from 3–6 independent experiments is shown.

To investigate whether the expression of CD155, the poliovirus receptor, on the surface of the different hematopoietic cell lines correlated with the capacity to propagate Sabin poliovirus type 1, human CD155 expression was determined using FACS analysis. In Figure 2 it can be seen that all hematopoietic cell lines, as well as Vero cells, express CD155. Vero, U937, and K562 had the highest level of expression, whereas the expression level of THP-1, KG1, and HL60 cells was lower. Also, not all HL60 cells were positive for CD155. Since Sabin poliovirus type 1 was not capable of replicating in THP1 and HL60 cells whereas replication in KG1 cells was efficient, expression levels of CD155 thus cannot fully predict the capacity of Sabin poliovirus type 1 to replicate in these cell lines.

### 3.3. Replication Kinetics of Sabin Type 1 in the Hematopoietic Tumor Cell Lines

**Figure 3:**
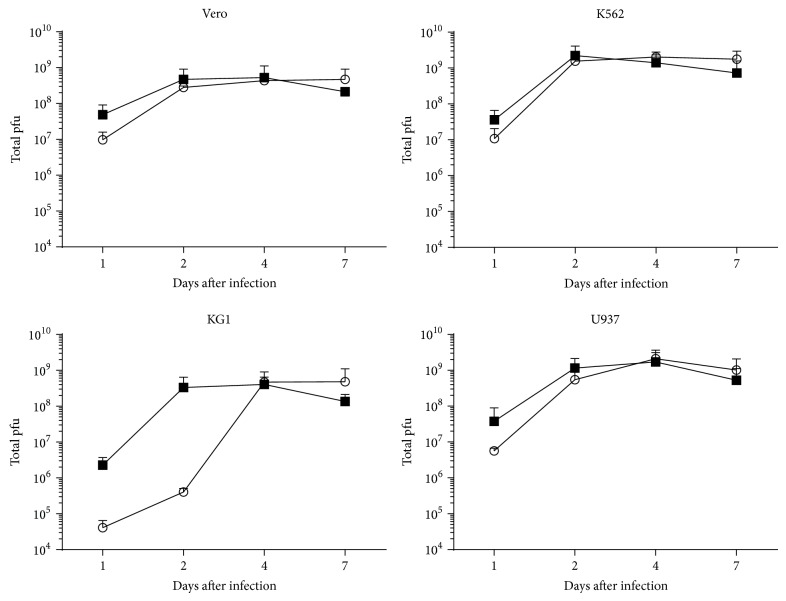
Replication kinetics of Sabin poliovirus type 1 (open circles) and adapted Sabin poliovirus type 1 that was serially passaged on Vero, K562, KG1, and U937 cells (black squares). Cells were infected with Sabin poliovirus type 1 from passage 0 or passage 5 and the titer in the supernatant was determined at days 1, 2, 4, and 7 after infection by endpoint dilution. The total amount of plaque forming units in these samples was determined and the mean ± SD from 3 independent experiments is shown.

To determine the timing of viral replication and viral release in the supernatant and to compare original with passaged virus, samples of the culture medium of cells infected with an MOI of 1 were taken at different time points after infection, and the virus titer in these samples was determined. The MOI of 1 was chosen in order to determine the kinetics of a single replication round and not to look at the effects of repeated infection cycles. Sabin poliovirus type 1 from the original stock (passage 0) was compared with the passaged virus (passage 5). In Figure 3 it can be seen that the titers in the supernatant are comparable between the parental and the passaged viruses in Vero, U937 and K562 cells, whereas in KG1 cells the passaged virus induced higher titers at days 1 and 2 after infection while the original virus needed 4 days to achieve comparable titers in the supernatant. This suggests that the virus passaged in the KG1 cells had an enhanced replication speed. In Vero, K562, and U937 cells a trend is observed that the virus titer in the supernatant at day 1 is slightly higher if cells are infected with the passaged virus compared to control virus, although the differences are not significant. It could be that in these cell lines also the replication speed of the passaged virus is slightly enhanced, but differences remain limited because after 2 days the maximal viral titer has been obtained, due to lysis of all the cells.

Furthermore, it can be concluded from these data that, for all three hematopoietic cell lines tested, the virus titer of the passaged virus at day 2 in the supernatant is comparable to the virus titer obtained 2 days after replication of Sabin poliovirus type 1 in Vero cells. Also, the virus titer in the culture medium remained stable during the 7 days of the experiment. Altogether, these data indicate that, with respect to the viral kinetics, all three human hematopoietic cancer cell lines are efficiently producing poliovirus particles.

### 3.4. High Titers of Sabin Type 1 after Infection of U937, KG1, or K562 with a Low MOI

**Figure 4:**
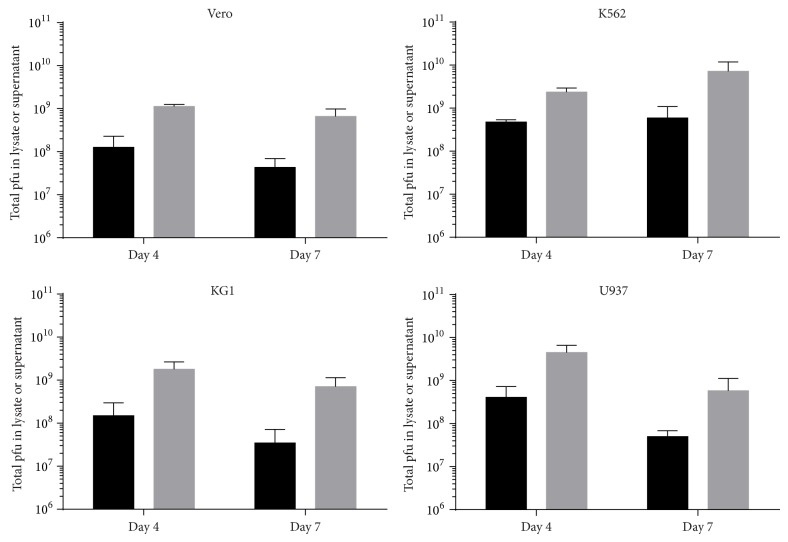
Infection of Vero, K562, KG1, or U937 at an MOI of 0.01 with passaged Sabin poliovirus type 1 on the various cell lines results in a high virus titer in the supernatant after 4 or 7 days. After infection, both cells (black) and supernatants (grey) were harvested at day 4 and day 7, and the virus titer was determined in these samples. The total amount of plaque forming units in these samples was determined and the mean ± SD from 3 independent experiments is shown.

For vaccine production purposes it is important that, after infection of cells with a low MOI, a high viral yield in the culture medium can be achieved. To investigate this, cells were infected with an MOI of 0.01 of passaged Sabin poliovirus type 1, and supernatant and cellular lysates were harvested separately at day 4 and day 7. The total viral titer in these samples was determined and is shown in Figure 4. In the supernatant of all cell lines tested, at day 4, a high virus titer, comparable to Sabin poliovirus type 1 replicated in Vero cells, was observed in the culture medium, indicating that virus replication was efficient during multiple rounds of replication. In K562 cells, the virus titer was moderately further increased at day 7 after infection, whereas in the other cell lines the virus titer at day 7 in the culture medium slightly decreased compared to the titer at day 4. Interestingly, at both day 4 and day 7 after infection, a high amount of virus was still present in the viable cells. This was also observed in Vero cells, and it suggests that not all cells were lysed at day 4 or day 7 by Sabin poliovirus type 1.

### 3.5. Efficient Replication of K562 Cells Grown in Cultibags with Sabin Poliovirus Type 1

**Figure 5:**
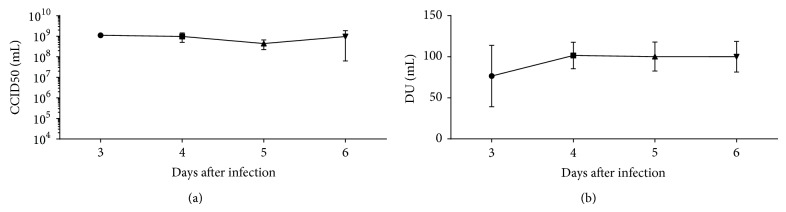
Efficient replication of Sabin poliovirus type 1 in K562 cells grown in Cultibags. K562 cells were infected with an MOI of 0.01 of passaged Sabin poliovirus type 1 when cells reached a concentration of 1.2 × 10^6^ cells/mL and samples of the supernatant were taken daily. In (a) and (b) the CCID50/mL and the D-antigen/mL at the various time points are shown, respectively. The mean ± SD from 3 independent experiments is shown.

To determine whether it is possible to produce Sabin poliovirus type 1 at a larger scale, K562 cells were grown in Cultibags. Cells were inoculated at a concentration of 0.3 × 10^6^ cells/mL in 1 L culture medium. After 4 days, in which the K562 cells grew to a concentration of 1.2–1.4 × 10^6^ cells/mL, the cells were infected with the passaged Sabin poliovirus type 1 at an MOI of 0.01. At days 3, 4, 5, and 6, the cell viability was determined and samples of the culture medium were taken, in which the virus titer and D-antigen concentration were determined. As can be seen in Figure 5(a), the virus replicated efficiently, resulting in titers of 1 × 10^9^ CCID50/mL in the culture medium already at day 3 after infection, which remained stable for at least the following 3 days. The D-antigen level at day 3 after infection (Figure 5(b)) was somewhat lower than that at later time points. Also, a large variation in the D-antigen level between the 3 experiments was observed at day 3, suggesting that not all viruses expressed D-antigen yet. However, at days 4, 5, and 6 after infection, D-antigen levels were high and comparable in all three experiments.

### 3.6. Surface Expression of CD54, CAR, and CD81 by All Hematopoietic Tumor Cell Lines

**Figure 6:**
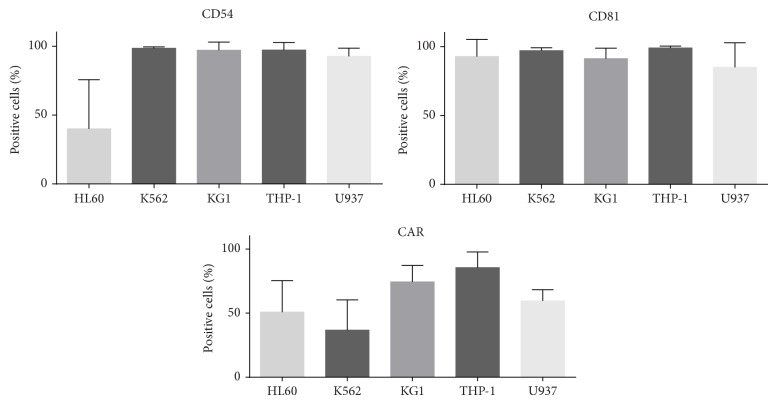
Surface expression of CD54, CAR, and CD81, the receptors for rhinovirus, coxsackie, adenovirus, and hepatitis C virus, on the hematopoietic tumor cell lines. The mean percentage of expressing cells ± SD from 3–6 independent experiments is shown.

To investigate whether the human suspension cell lines express multiple viral receptors, surface staining of CD54, CAR, and CD81 was performed. CD55, CAR, and CD81 are the receptors for rhinovirus, coxsackie, adenovirus, and hepatitis C virus, respectively. In Figure 6, the percentage of cells expressing the specific receptor is shown. All cell lines, except HL60, highly expressed CD54 and CD81. HL60 cells did express high levels of CD81, but CD54 was only expressed by 50% of the cells. With respect to CAR expression, differences between the cell lines were observed. A minority of K562 cells expressed CAR, whereas, for the other cell lines tested, 50–90% of the cells did express CAR. Whether the expression of these receptors predicts the capacity of the virus to replicate in these cells remains to be determined.

## 4. Discussion

High vaccination rates have helped to prevent many infectious diseases and resulted in less sickness and millions of lives saved (reviewed by Rappuoli et al.) [38]. Among the greatest success stories are the eradication of smallpox virus, rinderpest, and the polio eradication program, where poliovirus type 2 was already eradicated in 1999 [39–41]. Complete eradication of poliovirus is, however, more difficult than what has been initially anticipated, because of persistence of wild type poliovirus transmission and recurring outbreaks in polio-free countries (reviewed by Wassilak et al.) [42]. The capacity to develop vaccines that induce efficient immune responses in a short period of time, together with a large global immunization rate, is thus essential to prevent viral outbreaks or further spread of viruses. In the last decades, many different cell lines have been used in vaccine production processes, and new cell lines are still being developed. Since regulatory views are changing with respect to the risks and benefits of cell lines (reviewed by Hess et al.) [7], it is important to compare different cell lines for their capacity to propagate specific viruses. With such an approach, using viruses that belong to different viral groups, it might be possible in the future to select producer cell lines based upon scientific understanding instead of trial and error, and timelines needed to produce viral vaccines might be shortened. In this study, we performed a first step for such a comparison, with a focus on human suspension cell lines. These cell lines have an infinite lifespan and this, together with the fact that these cells grow in suspension, could facilitate vaccine production processes. All cell lines described in this study have been used extensively in experimental research. The disadvantage of these cell lines, obviously, is that these cell lines consist of tumor cells that are grown in serum-containing medium and are currently thus not qualified as suitable vaccine substrates. However, with changing regulatory opinions, it is foreseen that continuous cell lines will be accepted for the production of viral vaccines in the (near) future. Until then, studies, like this, can be used to gain knowledge about the interaction of cell lines with viruses and/or the development of new producer cell lines that are approved for the production of viral vaccines.

Because of the experience that our group has with poliovirus production processes [43–45], it was decided to perform this study with Sabin poliovirus type 1 as the first model virus.

First, it was determined whether the five selected cell lines were susceptible for poliovirus infection. K562, KG1, and U937 cell lines appeared to be capable of supporting replication of Sabin poliovirus type 1, whereas HL60 and THP-1 were not. All five cell lines did express CD155, the receptor for poliovirus entry. This means that receptor expression does not predict the capacity of a virus to replicate in a cell. Possible explanations for the lack of replication in the CD155 expressing HL60 and THP-1 cells could be that (i) CD155 expressed on these cell lines is nonfunctional, (ii) the nonsusceptible cell lines lack the expression of a coreceptor on the cellular membrane, or (iii) HL60 and THP-1 express an intracellular viral restriction factor. Since the tested cell lines have the capacity to differentiate towards several end-stages and it has been shown for other viruses that the differentiation stage of a cell can affect the susceptibility for a virus [33–35], it would also be interesting to determine permissiveness of HL60 and THP-1 for Sabin type 1 after differentiation of the cells towards different end-stages.

In the literature it has been described that human blood cell lines were susceptible for infection with poliovirus [46], and that study demonstrated that well-differentiated human blood cell lines are more susceptible to cytopathic effects of poliovirus than the less-differentiated blood cell lines. From the selection of cell lines we used in this study, K562 cells were the least differentiated, but with respect to viral replication we did not observe a difference between K562 cells and the more differentiated cell lines.

Another study compared the replication capacity of Mahoney poliovirus on K562 and U937 cells to HeLa cells [47]. Compared to HeLa cells, poliovirus replicated less efficiently in both K562 and U937 cells yielding a 20-fold and a 50-fold reduced virus output. Finally, a study by Benton et al. showed differences between K562 clones in their response to poliovirus infection, because two out of four K562 cell lines were killed by the poliovirus, whereas in the two other cell lines persistent infections were established [48]. In our study, we did not observe a persistent infection of K562 cells, nor that the poliovirus replicated less efficiently in U937 cells or the less-differentiated K562 cells. The discrepancies between the observed replication characteristics in these studies with our study could possibly be explained by the fact that in our study the replication characteristics of passaged Sabin type 1 were studied, meaning that the virus has had 5 passages to adapt to the new cell line. We did, however, observe already after the first passage of Sabin type 1 in K562 or U937 cells a high virus titer. This was the amount of virus present in culture medium together with the virus in the cellular lysates, so the majority of the detected virus could have still been present in the cellular fraction, whereas the other studies determined the amount of virus present in the culture medium only. Another difference between these studies was that Benton et al. and Lopez-Guerrera et al. used Mahoney strain of type 1 poliovirus, and in our study attenuated Sabin poliovirus type 1 was used.

In two more recent papers, the replication capacity of Sabin types 1, 2, and 3 or wild type polioviruses was studied on a panel of different suspension cell lines, deriving from humans, avians, or canines. In both studies the virus was not adapted to the new cell lines, but experiments were directly performed with virus produced on Vero cells. Vlecken et al. compared a number of adherent and suspension cell lines for the capacity to replicate Sabin poliovirus types 1, 2, and 3. Of the 5 cell lines tested (BHK-21, CHO-K1, CAP, sMDCK, and AGE1.CR.HS), only the CAP cell line was capable of propagating Sabin type polioviruses, whereas all the other suspension cell lines were not capable of supporting viral replication [49]. In the second study, published by Sanders et al., the capacity of PER.C6 to support replication of Brunenders, MEF-1, and Saukett poliovirus was determined in comparison to Vero cells [50]. PER.C6 is an immortalized human cell line capable of supporting the replication of a number of viruses, like influenza virus and West Nile virus [51, 52]. The poliovirus was capable of replicating efficiently in the PER.C6 cell line, resulting in a high virus yield, which can be attributed to the fact that PER.C6 cells can be cultured under optimized conditions to very high cell densities. Altogether, these two studies suggest that, for efficient replication of poliovirus, human or primate cells are needed, since, also in the adherent cell lines tested by Vlecken et al., viral replication was not observed in cell lines with another origin [49]. This observation underscores the need of a wider panel of human/primate cell lines for the production of viral vaccines for human diseases. In our study, we have identified three additional cell lines that are susceptible for infection and replication of poliovirus that can also be grown at a larger scale in a disposable bioreactor system (only tested for K562 cells). It is important to realize that, by passaging a virus on a new cell line, the virus can adapt to this new cell line to optimize its replication cycle. This can also be accompanied by changes in antigenicity or virulence of the adapted virus. A new combination of cell line and virus should thus always be analyzed thoroughly, before the new cell line can be used for the production of viral vaccines. In this study, we did observe a difference in replication speed between original virus and virus passaged on KG1 cells, but sequencing is needed to determine whether the virus truly adapted to the cell line.

Since we already observed that also other Sabin poliovirus types are capable of replicating in the permissive three cell lines (Oosterhoff et al., unpublished data) and that all cell lines express other viral receptors, needed for efficient infection with rhinovirus, coxsackie, adenovirus, and hepatitis C virus, it would thus be interesting to determine the capacity of these viruses to replicate in these cell lines. Ultimately both the panel of (human) cell lines and viruses need to be expanded, in order to be able to predict upfront the suitability of cell lines for the production of specific viral vaccines, based on scientific understanding, thereby reducing timelines and costs in vaccine production processes.

## 5. Conclusion

In conclusion, these data demonstrate that K562, KG1, and U937 cell lines are efficient in supporting the replication of Sabin poliovirus type 1. All five human hematopoietic cell lines expressed CD155, which thus did not explain susceptibility to viral replication, although we did not study the functionality of CD155. Furthermore, we have shown that in KG1 cells the passaged Sabin poliovirus type 1 replicated more rapidly than the control virus. Infection of K562, KG1, and U937 at an MOI of 0.01 resulted in a high viral titer in the culture medium after 4 days. Also, K562 cells grown and infected with adapted Sabin poliovirus type 1 in a disposable bioreactor system yielded high viral titers in the culture medium. Altogether, it is concluded that K562, KG1, and U937 cell lines can be used for the propagation of poliovirus and in follow-up studies the capacity of other (entero)viruses to replicate in these cell lines will be determined.
